# The Synergistic Cytotoxic Effects of GW5074 and Sorafenib by Impacting Mitochondrial Functions in Human Colorectal Cancer Cell Lines

**DOI:** 10.3389/fonc.2022.925653

**Published:** 2022-06-07

**Authors:** Je-Ming Hu, Yung-Lung Chang, Cheng-Chih Hsieh, Shih-Ming Huang

**Affiliations:** ^1^Institute of Medical Sciences, National Defense Medical Center, Taipei, Taiwan; ^2^Department of Surgery, Division of Colorectal Surgery, Tri-Service General Hospital, National Defense Medical Center, Taipei, Taiwan; ^3^Department of Biochemistry, National Defense Medical Center, Taipei, Taiwan; ^4^School of Pharmacy and Institute of Pharmacy, National Defense Medical Center, Taipei, Taiwan; ^5^Department of Pharmacy, Kaohsiung Veterans General Hospital, Kaohsiung, Taiwan

**Keywords:** colorectal cancer, C-RAF inhibitor, multi-kinase inhibitor, mitochondrial dysfunction, the combination index

## Abstract

Colorectal cancer (CRC) ranks third in the United States for incidence or mortality. Surgical resection is the primary treatment for patients at an early stage, while patients with advanced and metastatic CRC receive combined treatment with chemotherapy, radiotherapy, or targeted therapy. C-RAF plays a key role in maintaining clonogenic and tumorigenic capacity in CRC cells and it might be a potential therapeutic target for CRC. Sorafenib is a popular oral multi-kinase inhibitor, including a B-RAF inhibitor that targets the RAF-MEK-ERK pathway. Sorafenib, as a single agent, has tumor-suppressing efficacy, but its clinical application is limited due to many complex drug resistance mechanisms and side effects. GW5074 is one of the C-RAF inhibitors and has the potential to enhance the efficacy of existing cancer chemotherapies. In this study, we investigated whether the combination of sorafenib with GW5074 could reduce the dosage of sorafenib and enhance its tumor-suppressive effect in two CRC cell lines, HCT116 and LoVo cells. Our findings demonstrate that GW5074 can potentiate the cytotoxicity of sorafenib and dramatically reduce the half-maximal inhibitory concentration (IC50) dose of sorafenib from 17 and 31 µM to 0.14 and 0.01 µM in HCT116 and LoVo cells, respectively. GW5074, similar to sorafenib, suppressed the cellular proliferation and induced cellular apoptosis and cytosolic ROS, but had no further enhancement on the above-mentioned effects when combined with sorafenib. The synergistic effects of GW5074 and sorafenib were mainly found in mitochondrial functions, including ROS generation, membrane potential disruption, and fission–fusion dynamics, which were examined by using the flow cytometry analysis. In summary, the C-RAF inhibitor GW5074 might potentiate the cytotoxicity of the B-RAF inhibitor sorafenib mediated through mitochondrial dysfunctions, suggesting that GW5074 potentially serves as a sensitizer for sorafenib application to reduce the risk of drug resistance of CRC treatment. Our findings also provide novel insights on using C-RAF inhibitors combined with sorafenib, the current CRC therapeutic drug choice, in CRC treatment.

## Introduction

In the 2022 annual cancer statistics report of the American Cancer Society, colorectal cancer (CRC) ranks third in the United States for incidence or mortality, regardless of gender (1). Genetic and environmental factors contribute significantly to the etiology of CRC, including family history, smoking, alcohol intake, obesity, diabetes, inflammatory bowel disease, etc. (2). Surgical resection is the primary treatment for patients at an early stage, while patients with advanced and metastatic CRC (mCRC) are treated with chemotherapy such as 5-fluorouracil, oxaliplatin, etc., combined with radiotherapy and targeted therapy (3). However, in 25% of patients with advanced mCRC, the surgical effect is very limited. The efficacy of chemotherapy may also be reduced by the development of drug resistance and cancer recurrence. Therefore, a new drug or combinatory therapy for CRC treatment, especially mCRC, is urgently needed to improve its overall survival rate.

Since CRC is a disease that accumulates multiple genetic mutations in the epithelial tissue of the colon and rectum, molecular biomarkers play an important role in the individualized treatment of CRC patients (4). Using these biomarkers such as RAS, B-RAF, and microsatellite instability status, the prognosis for CRC patients can be stratified, and more precise adjuvant treatment plans can be provided (5). Activation of mitogen-activated protein kinase (MAPK) pathways that regulate multiple cellular activities including proliferation, differentiation, and apoptosis have been identified as the critical oncogenic mechanisms in CRC (6). RAF kinases (A-RAF, B-RAF, and C-RAF), which play an integral role in this pathway, are regulated through a network of protein–protein interactions and phosphorylation–dephosphorylation events (7). B-RAF is the family member most easily activated by RAS because both A-RAF and C-RAF need additional steps to reach maximal activation. In addition, B-RAF (V600E) mutation is a driver mutation—it constitutively activates the MAPK/extracellular signal-regulated kinase (ERK) kinase (MEK)-ERK signaling pathway downstream of KRAS (8), which is present in 5–15% of CRC (9).

The contribution of RAF to the hallmarks and phenotypes of cancer is reported. Three types of RAF enforce the dimerization of endogenous RAFs, such as B-RAF with C-RAF or A-RAF. RAF kinases are prime targets for the design and application of molecule-target therapies for cancers, including melanoma, renal cancer cells, and hepatocellular carcinoma. Cancer cells develop chemo-resistance by several different molecular mechanisms involving the activation of other MEK kinases, but also the upregulation of receptor tyrosine kinases and other pathways downstream from RAS (10). The underlying mechanism is an allosteric effect of the RAF inhibitors, the paradoxical increase in the proliferation and activation of the MEK–ERK pathway in cells. Until now, RAF is still a fascinating topic for basic and clinical researchers.

Sorafenib is an oral multi-kinase inhibitor that targets the RAF-MEK-ERK pathway, which can promote apoptosis and reduce angiogenesis and inhibit tumor cell proliferation (11–13). Furthermore, it modulates the RAF-MEK-ERK pathway by inhibiting C- and B-RAF, thereby affecting tumor cell proliferation, even in KRAS-mutated cancers (14). Sorafenib has recently been used as a targeted treatment for mCRC patients, where its therapeutic value has been recognized (15, 16). However, while sorafenib has tumor-suppressing efficacy as a single agent, its clinical application is limited by many complex drug resistance mechanisms and side effects. C-RAF plays a key role in maintaining clonogenic and tumorigenic capacity in CRC cells (containing KRAS mutations), and it might be a potential therapeutic target for CRC (17). GW5074 is one of the C-RAF inhibitors which are broad-spectrum antitumor agents and have the potential to enhance the efficacy of existing cancer chemotherapies (18, 19). Despite their potency, C-RAF inhibitors lack relative therapeutic efficacy due to poor bioavailability.

A recent study demonstrated the effectiveness of the combination of sorafenib and GW5074 for renal cancer cells, targeting C-RAF for the regulation of its mitochondrial localization and function involved in cell death cascades (20). Here, we investigated whether the combination of sorafenib with GW5074 could reduce the dosage of sorafenib and enhance its tumor-suppressive effect in two CRC cell lines, HCT116 and LoVo cells. Our findings also provide novel insights on using C-RAF inhibitors in CRC treatment.

## Materials and Methods

### Cell Culture and Chemical

HCT116 and LoVo cells were cultured in Dulbecco’s modified Eagle’s medium (DMEM) containing 10% fetal bovine serum (FBS) and 1% penicillin streptomycin (Invitrogen, CA, USA) at 37°C and 5% CO_2_. GW5074 (3-(3,5-Dibromo-4-hydroxy-benzylidene)-5-iodo-1,3-dihydro-indol-2-one), sorafenib, and 2’,7-dichlorofluorescein diacetate (DCFH-DA) were obtained from Sigma-Aldrich (MO, USA).

### Metabolic Activity Analysis and the Combination Index

MTS (3-(4,5-dimethylthiazol-2-yl)-5-(3-carboxymethoxyphenyl)-2-(4-sulfophenyl)- 2H-tetrazolium) assay was performed using the CellTiter 96 Aqueous One Solution Cell Proliferation Assay kit (Promega, WI, USA). Briefly, HCT116 and LoVo cells were seeded onto 96-well plates and cultured in the presence of the indicated drugs for 24 h. The cells were then incubated with MTS solution (20 μl/well) for 2 h at 37°C, and the absorbances at 490 nm were measured using an ELISA plate reader (Multiskan EX, Thermo, MA, USA). The relative metabolic activity was calculated based on the absorbance ratio between cells cultured with the indicated drugs and the vehicle control, which were assigned a value of 100.

The combination index (CI) was calculated utilizing CalcuSyn (Biosoft, Cambridge, UK) to generate the isobolograms for the determination of synergistic, additive, and antagonistic combinatory effect. Typically, a CI value <1 denotes a synergistic combination effect, and a CI value >1 denotes an antagonistic combination effect (21).

### Fluorescence-Activated Cell Sorting, Cell Cycle Profiling, and Cellular Proliferation Analyses

For cell cycle profile and cellular proliferation, we performed BrdU/7-AAD analysis with the FITC BrdU Flow Kit (BD Biosciences, CA, USA) according to the manufacturer’s instructions. Cell cycle profiles were measured according to cellular DNA content using FACS. Cells were fixed in 70% ice-cold ethanol, stored at −30°C overnight, washed two times with ice-cold PBS supplemented with 1% FBS, and then stained with 7-AAD (7-Aminoactinomycin D). The percentage of positive HCT116 and LoVo cells was determined using flow cytometry. All samples were analyzed using a FACSCalibur flow cytometer (BD Biosciences). Data were analyzed using Cell Quest Pro software (BD Biosciences). Procedural details were described previously (22, 23).

### Apoptosis and ROS Assays

To evaluate the incidence of apoptosis, we used the PE Annexin V Apoptosis Detection Kit according to the manufacturer’s instructions (BD Biosciences). Apoptotic cells were then analyzed using flow cytometry. To detect the production of ROS, we plated cells in 6-well plates and treated GW5074 and sorafenib. After 24 h of drug treatment, living cells were stained with 20 μM DCFH-DA (Sigma-Aldrich) and incubated at 37°C for 1 h. Stained cells were determined using flow cytometry.

### Mitochondrial ROS Assay

The fluorescent marker MitoSOX™Red (Invitrogen) was used to determine mitochondrial ROS levels. Cells were incubated for the indicated times with different combinations of GW5074 and sorafenib. Living cells were then stained with 5 µM MitoSOX™Red and harvested for 10 min at 37°C. After washing the cells once with PBS, they were determined using flow cytometry.

### Mitochondrial Fission–Fusion Transient Analysis

HCT116 and LoVo cells were seeded onto 6-well plates. After treating the cells for the indicated times with selected combinations of GW5074 and sorafenib, the cells were washed, incubated with 100 nM MitoView™Green (Biotium, CA, USA) at 37°C for 15 min, and washed again three times with PBS. They were determined using flow cytometry.

### Mitochondrial Membrane Potential Analysis

Mitochondrial depolarization was measured as a function of a decrease in the red/green fluorescence intensity ratio. All dead and viable cells were harvested, washed with PBS, and incubated with 1× binding buffer containing the MMP-sensitive fluorescent dye JC-1 for 30 min at 37°C in the dark. After washing the cells once with PBS, JC-1 fluorescence was analyzed on channels FL-1 and FL-2 of the FACSCalibur flow cytometer using Cell Quest Pro software (BD Biosciences) to detect monomer (green fluorescence) and aggregate (red fluorescence) forms of the dye, respectively. The cell volume gating strategy involved forward scatter height (FSC-H) and side scatter height (SSC-H), and the median fluorescence intensity of the vehicle was used as the starting point for M2 gating.

### Western Blotting

HCT116 and LoVo cells were lysed in radioimmunoprecipitation assay buffer (100 mM Tris-HCl (pH 8.0), 150 mM NaCl, 0.1% SDS, and 1% Triton X-100) at 4°C. Proteins in the resultant lysates were separated by sodium dodecyl sulfate-polyacrylamide gel electrophoresis and analyzed by immunoblotting with antibodies against ACTN (sc-17829, mouse), ATF1 (sc-243, mouse), ATF3 (sc-81189, mouse), ATF4 (sc-390063, mouse), ATF5 (sc-377168, mouse), catalase (sc-271803, mouse), DRP-1 (sc-101270, mouse), Mfn1 (sc-166644, mouse), Nrf2 (sc-365949, mouse), p62 (sc-28359, mouse), Parkin (sc-133167, mouse), PGC-1α (sc-518052, mouse), SOD1 (sc-101523, mouse), SOD2 (sc-133134, mouse), SOD3 (sc-377168, mouse), mtTFA (sc-376672, mouse), TFEB (sc-166736, mouse), Tom20 (sc-17764, mouse) (Santa Cruz Biotechnology, CA, USA), HO-1 (ADI-SPA-895-F, rabbit, Enzo life sciences, NY, USA), γ.H2AX (ab81299, rabbit, Abcam, Cambridge, UK), GAPDH (60004-1-1g, mouse), α-Tubulin (13730-1-AP, mouse) (Proteintech, IL, USA), XBP1 (NBP1-77253, rabbit), ATF6 (NBP1-40256, mouse) (Novus, CO, USA), AMPK (2535, rabbit), p-AMPK (5831, rabbit), Caspase 3 (9662, rabbit), CHOP (2895, mouse), p70S6K (2708, rabbit), p-p70S6K (9205, rabbit). p-DRP-1 (3455, rabbit), eIF2α (9722, rabbit), p-eIF2α (9721, rabbit), LC3B (2775, rabbit), and PARP (9542, rabbit) (Cell Signaling Technology, MA, USA). Thereafter, the blots were incubated with horseradish peroxidase-conjugated secondary antibody (Santa Cruz Biotechnology). The immunoreactive proteins were detected using ECL™ Western Blotting Detection Reagent and Amersham Hyperfilm™ ECL (GE Healthcare, IL, USA).

### RNA Extraction and Reverse Transcription PCR

HCT116 and LoVo cells were lysed by TRIzol reagent (Invitrogen) to isolate total RNAs. Reverse transcription for first-strand cDNA synthesis was conducted using MMLV reverse transcriptase (Epicenter Biotechnologies, WI, USA) with 1 µg of total RNA for 60 min at 37°C. PCR reactions were operated on a Veriti Thermal Cycler (Applied Biosystems, MA, USA). Primers and the number of PCR reaction cycles used were listed in **Table 1**.

**Table 1:** Primers were used in this study.

Gene name	Primer sequence (Forward)	Primer sequence (Reverse)	Cycle #
ATF1	5’-GCTCAACAGGTATCATCTTTATCAG-3’	5’-accacagtttgtggcagaga-3’	30
ATF3	5’-GAGGATTTTGCTAACCTGAC-3’	5’-TAGCTCTGCAATGTTCCTTC-3’	28
ATF4	5’-TTCCAGCAAAGCACCGCAAC-3’	5’-AGGGCATCCAAGTCGAACTCCT-3’	30
ATF5	5’-AAGTCGGCGGCTCTGAGGTA-3’	5’-GGACTCTGCCCGTTCCTTCA-3’	30
ATF6	5’-ATGAAGTTGTGTCAGAGAAC-3’	5’-GGGTGCTATTGTAATGACTCA-3’	30
CHOP	5’-CATTGCCTTTCTCCTTCGGG-3’	5’-GCCGTTCATTCTCTTCAGCT-3’	30
eIF2a	5’-ACCTCAGAATGCCGGGTCTA-3’	5’-GTGGGGTCAAGCGCCTATTA-3’	28
XBP1	5’-CCTTGTAGTTGAGAACCAGG-3’	5’-GGGGCTTGGTATATATGTGG-3’	30
TOM20	5’-GTGTATGCGGGGCCCTTTTC-3’	5’-ACATCATCTTCAGCCAAGCTCT-3’	28
mtTFA	5’-GCGTTTCTCCGAAGCATGTG-3’	5’-TTGTGCGACGTAGAAGATCC-3’	30
PGC-1α	5’-GTGTCACCACCCAAATCCTTA-3’	5’-ATTCTTCCCTCTTCAGCCTCT-3’	35
Parkin	5’-AAGGAGGTGGTTGCTAAGCGAC-3’	5’-CTGGGTCAAGGTGAGCGTTGC-3’	35
TFEB	5’-GGTGTTGAAGGTGCAGTCC-3’	5’-GGGTAGCGTGTTGGGCATCTG-3’	30
AMPK	5’-CGGCAAAGTGAAGGTTGGC-3’	5’-TCCTTCGTGGAGCCTGTTTT-3’	30
p70S6K	5’-GGAGCCTGGGAGCCCTGATGTA-3’	5’-GAAGCCCTCTTTGATGCTGTCC-3’	30
18S	5’-CAGCCACCCGAGATTGAGCA-3’	5’-TAGTAGCGACGGGCGGTGTG-3’	32

### Immunofluorescent Staining

Cells were incubated for 24 h on glass coverslips and then fixed for 5 min in 10% formaldehyde in PBS, permeabilized with 0.1% Triton X-100 in PBS, blocked with 1% BSA (Sigma, Burlington, MA, USA) in PBS, and incubated with mouse anti-human PGC-1a (Santa Cruz Biotechnology) or mouse anti-human Tom20 (Santa Cruz Biotechnology) in 1% BSA overnight at 4°C. Thereafter, the cells were washed three times with PBS and incubated with Alexa 488-goat anti-mouse IgG secondary antibody (1:400; ThermoFisher Scientific, Waltham, MA, USA) in 1% BSA for1 h at room temperature. After three more washes with PBS for 5 min each, the nuclei were stained with DAPI (Sigma, Burlington, MA, USA) for 5 min at room temperature. Finally, the coverslips were mounted on glass slides with a mounting medium (ThermoFisher Scientific, Waltham, MA, USA) and examined under a Leica Thunder microscope.

### Statistical Analysis

Values were expressed as the mean ± SD of at least three independent experiments. All comparisons between groups (vehicle and drug) were conducted using Student’s *t*-test. Statistical significance was set at *p* < 0.05.

## Results

### The Cytotoxicity of Sorafenib and GW5074 and Their Combinatory Effect on HCT116 and LoVo Cell Lines

**Figure 1:**
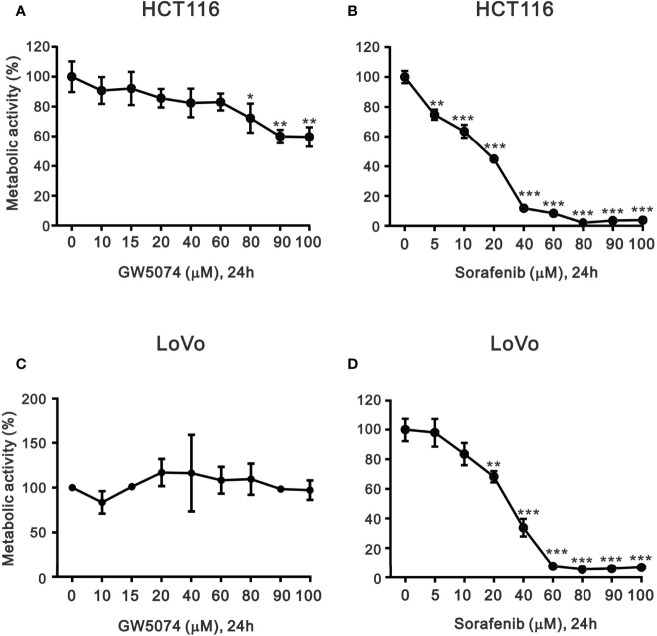
The effects of GW5074 and sorafenib on the cytotoxicity of HCT116 and LoVo cells. HCT116 and LoVo cells **(A, C)** were treated with 0, 10, 15, 20, 40, 60, 80, and 100 µM GW5074 for 24 h; **(B, D)** were treated with 0, 5, 10, 20, 40, 60, 80, 90, and 100 µM sorafenib for 24 h. Metabolic activity was measured using MTS assays. The results are representative of three independent experiments. *p < 0.05, **p < 0.01, and ***p < 0.001.

**Figure 2:**
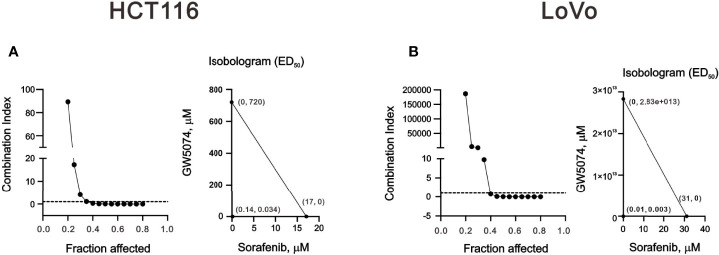
The combination index of GW5074 and sorafenib in HCT116 and LoVo cells. HCT116 **(A)** and LoVo **(B)** cells were treated with 0, 0.313, 0.625, 1.25, 2.5, 5, 10, and 20 µM sorafenib for 24 h and 0, 0.625, 1.25, 2.5, 5, 10, 20, and 40 µM GW5074 for 24 h. Metabolic activity was measured using MTS assays. The combination index of sorafenib plus GW5074 in HCT116 **(A)** and LoVo **(B)** cells. Isobolograms (ED_50_) of sorafenib or GW5074 were calculated using CalcuSyn software.

To explore the effects of two RAF inhibitors, B-RAF inhibitor sorafenib, and C-RAF inhibitor GW5074, on the human CRC cell lines, HCT116 and LoVo cells, the changes in cell viability were first determined using the MTT analysis. The results indicated that a significant decrease in metabolic activity was observed in HCT116 and LoVo cells with sorafenib treatment (**Figure 1**). Moreover, despite the higher dose of GW5074, the cell viability of HCT116 was still approximately 60% (**Figure 1A**), while LoVo cells did not respond at all (**Figure 1C**). Both HCT116 and LoVo cells responded well at a higher dosage of Sorafenib (**Figures 1B, D**). We further examined whether GW5074 had a synergistic effect on cell viability with sorafenib on HCT116 and LoVo cells using the combination index analysis. When the combination index score is less than 1, it means there is a synergistic effect, and when the score is greater than 1, it means there is an antagonistic effect. The results showed that combined treatment had a synergistic effect in HCT116 and LoVo cells, both of which could dramatically reduce the IC50 dose of sorafenib from 17 and 31 µM to 0.14 and 0.01 µM at the dosage of GW5074 0.034 and 0.003 µM, respectively (**Figure 2**).

### The Functional Interactions Between Sorafenib and GW5074 in the Cell Cycle, Cellular Apoptosis, and Cellular Proliferation

**Figure 3:**
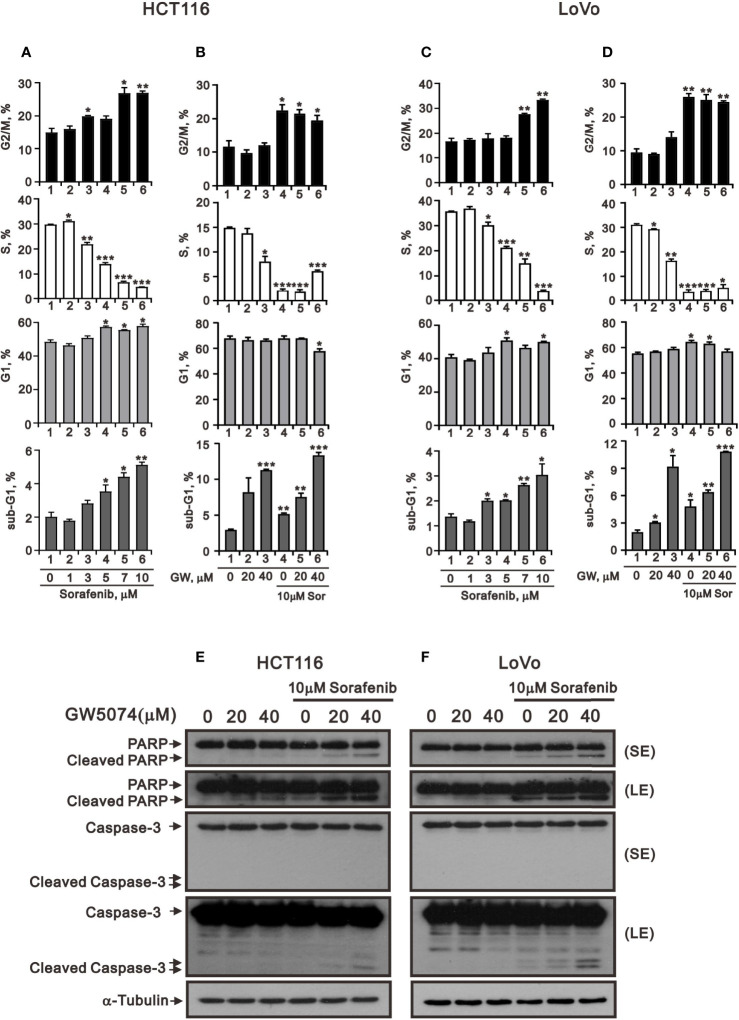
The effects of GW5074 and sorafenib on the cell cycle profile of HCT116 and LoVo cells. HCT116 **(A)** and LoVo **(C)** cells were treated with 0, 1, 3, 5, 7, and 10 µM sorafenib for 24 h. HCT116 **(B)** and LoVo **(D)** cells were treated for 24 h with 0, 20, and 40 µM GW5074 in the absence or presence of 10 µM sorafenib. **(E, F)** Western blot analysis was applied for the cleavage off PARP and Caspase 3 proteins. α-tubulin is a loading control protein. SE: shorter exposure; LE: longer exposure. The results (A–D) are representative of three independent experiments. *p < 0.05, **p < 0.01, and ***p < 0.001.

**Figure 4:**
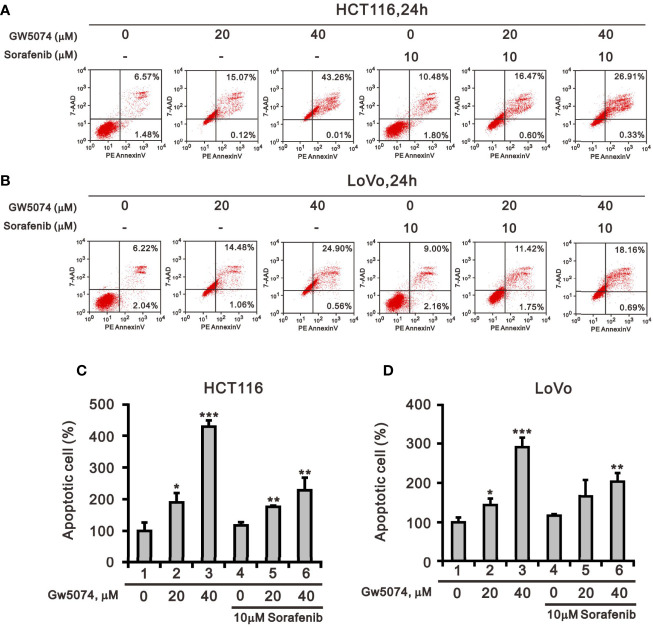
The effects of GW5074 and sorafenib on cellular apoptosis of HCT116 and LoVo cells. HCT116 **(A)** and LoVo **(B)** cells were treated with 0, 20, and 40 µM GW5074 in the absence or presence of 10 µM sorafenib for 24 h. They were then subjected to Annexin V apoptosis analysis. Early apoptotic cells are PE Annexin V-positive and 7-AAD-negative, while late apoptotic cells are both PE Annexin V- and 7-AAD-positive. HCT116 **(C)** and LoVo **(D)** cells were measured the percentage (early apoptosis plus late apoptosis) compared with vehicle alone (100%). The results are representative of three independent experiments. *p < 0.05, **p < 0.01, and ***p < 0.001.

**Figure 1** showed that GW5074 consistently potentiates the cytotoxicity of sorafenib in CRC cells, similar to our previous finding in renal carcinoma cells (20). Next, we explored the effects of combined treatment on different cellular mechanisms of CRC cells, including the cell cycle profile, cellular apoptosis, cellular proliferation, and ROS generation. To determine the functional roles of sorafenib, GW5074, and both combined in the cell cycle profile, we treated HCT116 and LoVo cells with indicated amounts of sorafenib (0, 1, 3, 5, 7, and 10 μM), GW5074 (0, 20, and 40 μM), and both combined (0, 20, and 40 μM GW5074 combined with 10 μM sorafenib). Sorafenib increased the populations of subG1, G1, and G2/M phases and decreased the population of the S phase in a dose-dependent manner (**Figures 3A, C**). GW5074 increased the population of the subG1 phase and decreased the population of the S phase in a dose-dependent manner (**Figures 3B, D**). The combined results indicate that GW5074 failed to potentiate the effects of sorafenib on the cell cycle profile, except for the population of the subG1 phase in the highest dose combination (**Figures 3B, D**). The cleavage forms of PARP and caspase 3 are biomarkers for cellular apoptosis (24). Hence, we further examined the cleaved trends of PARP and caspase 3 to confirm the increase in the population of subG1 in HCT116 and LoVo cells. We observed increasing amounts of cleaved PARP and caspase 3 by sorafenib combined with increasing amounts of GW5074 (**Figures 3E, F**). In the Annexin V-PE/7-AAD double fluorescence staining apoptosis analysis, our results demonstrated that sorafenib, GW5074, and a combination of both did not affect the early apoptosis stage in HCT116 and LoVo cells (**Figure 4**). We detected the apparent effects on the late apoptosis stages treated with sorafenib and GW5074. However, both combinations revealed that GW5074 failed to enhance the effect of sorafenib because of the dramatic induction by GW5074 itself.

**Figure 5:**
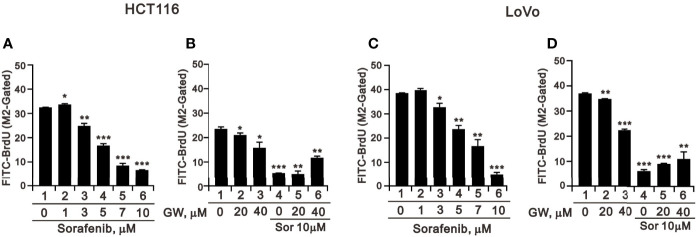
The effects of GW5074 and sorafenib on cellular proliferation of HCT116 and LoVo cells. HCT116 **(A)** and LoVo **(C)** cells were treated with 0, 1, 3, 5, 7, and 10 µM sorafenib for 24 h. HCT116 **(B)** and LoVo **(D)** cells were treated for 24 h with 0, 20, and 40 µM GW5074 in the absence or presence of 10 µM sorafenib. They were subjected to BrdU proliferation analysis. The results are representative of three independent experiments. *p < 0.05, **p < 0.01, and ***p < 0.001.

We applied the BrdU analysis for cellular proliferation to confirm the suppressive effect of sorafenib on the population of the S phase in HCT116 and LoVo cells (**Figures 3A–D**). Sorafenib suppressed cellular proliferation in a dose-dependent manner and its more suppressive effects than GW5074 in HCT116 and LoVo cells (**Figure 5**). GW5074 failed to further suppress the sorafenib-suppressed cellular proliferation in HCT116 and LoVo cells. However, in the combined treatment, GW5074 failed to potentiate the suppressive effect of 10 μM sorafenib on the cellular proliferation capacity in both cell lines (**Figures 5B, D**).

### The Cytotoxic Effects of Combined Sorafenib and GW5074 on the Endoplasmic Reticulum Stress and the ROS Status in HCT116 and LoVo Cells

**Figure 6:**
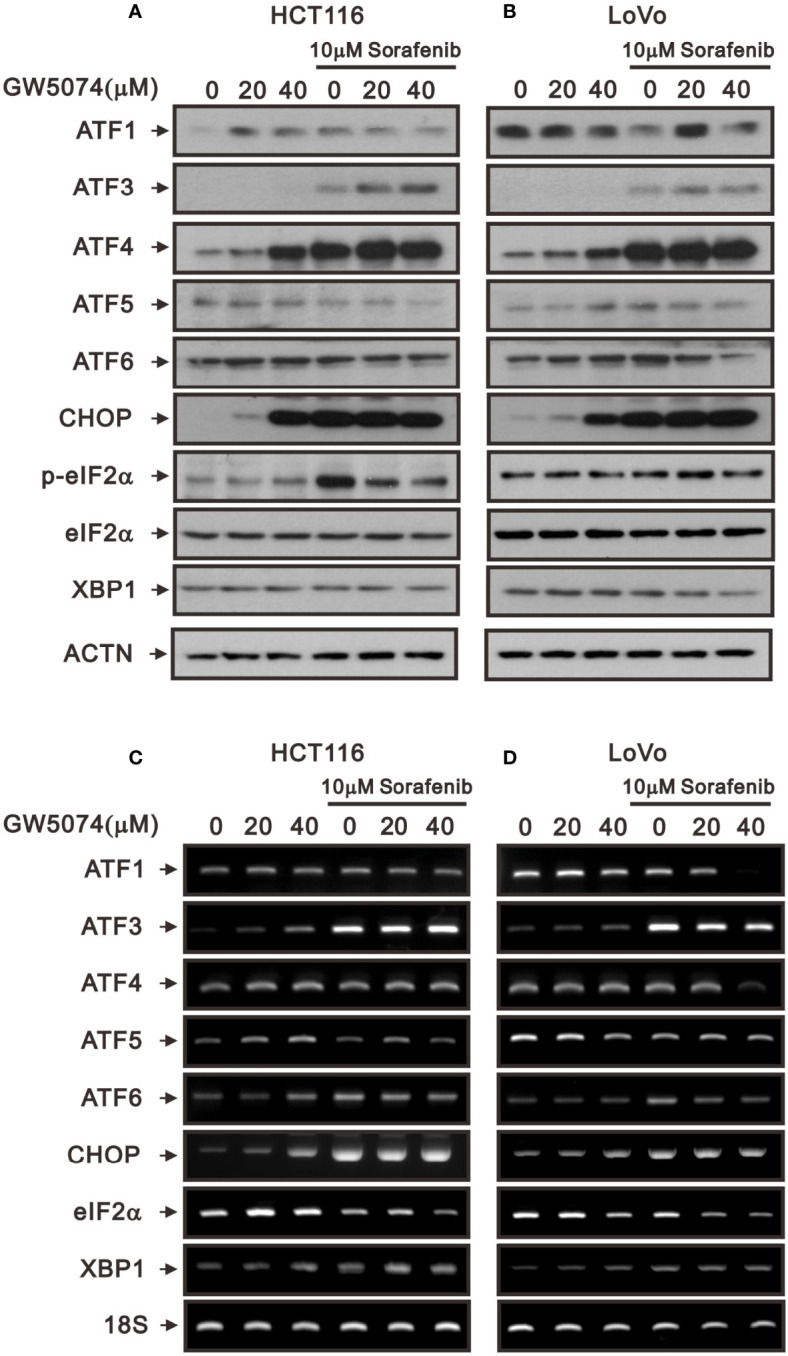
The effects of GW5074 and sorafenib on ER stress of HCT116 and LoVo cells. HCT116 and LoVo cells were treated with 0, 20, and 4 µM GW5074 in the absence or presence of 10 µM sorafenib for 24 h. **(A, B)** Cell lysates were subjected to Western blotting analysis. ACTN is a loading control protein. **(C, D)** Total RNA were subjected to RT-PCR analysis. 18S is a loading control.

Tyrosine kinase inhibitors can result in cytotoxicity mediated through the pathways of endoplasmic reticulum (ER) stress, autophagy, and oxidative stress (25, 26). To deal with the ER stress response, cells activate a series of signaling pathways, including PKR-like ER kinase (PERK), inositol-requiring transmembrane kinase/endoribonuclease 1α (IRE1α), and activating transcription factor 6 (ATF6) pathways, termed the unfolded protein response (UPR), which can either be protective (usually in the short term) or detrimental (usually in the long term). Here, we examined ER stress-related proteins and the consistent increasing trends of ATF3, ATF4, and CHOP in HCTA116 and LoVo cells treated with sorafenib, GW5074, and both combined using the western blotting analysis (**Figures 6A, B**). ATF5 and ATF6 had consistent increasing trends in HCTA116 and LoVo cells treated with individual sorafenib and GW5074 but had decreasing trends with both combined (**Figures 6A, B**). The decreased trend by both combined was observed in the ratio of p-eIF2/eIF2 and XBP-1, even sorafenib elevated the p-eIF2/eIF2 ratio in HCT116 cells. We further examined these above effects on related mRNAs using the RT-PCR analysis (**Figures 6C, D**). ATF3 and CHOP mRNAs were consistently increasing trend with their proteins in HCTA116 and LoVo cells. ATF4 mRNA was inconsistent with its protein-increasing trend. Other mRNAs were hard to make clear relation between protein and mRNA expression.

**Figure 7:**
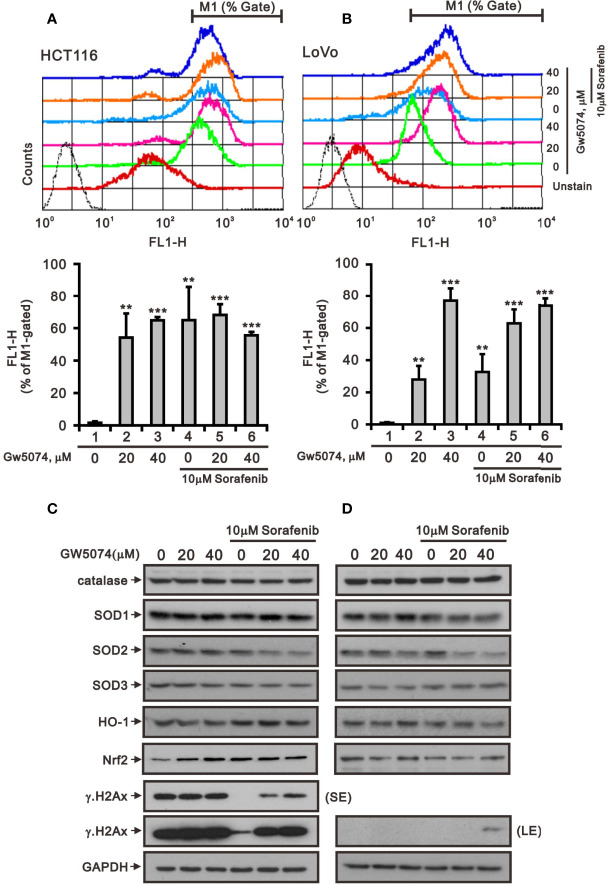
The effects of GW5074 and sorafenib on the cytosolic ROS generation of HCT116 and LoVo cells. HCT116 **(A)** and LoVo **(B)** cells were treated with 0, 20, and 40 µM GW5074 in the absence or presence of 10 µM sorafenib for 24 h, after which the live cells were stained with 20 μM DCFH-DA for 10 min at 37°C and assayed using a flow cytometer. **(C, D)** Cell lysates were subjected to Western blotting analysis with antibodies against SOD 1-3, Nrf2, HO-1, and γH2A.x. GAPDH is a loading control protein. SE, shorter exposure; LE, longer exposure. The results **(A, B)** are representative of three independent experiments. **p < 0.01 and ***p < 0.001.

**Figure 8:**
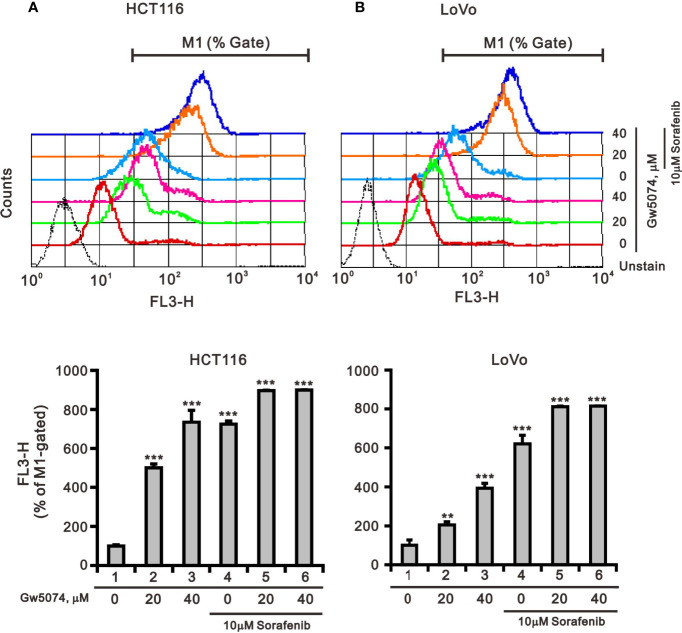
The effects of GW5074 and sorafenib on the mitochondrial ROS generation of HCT116 and LoVo cells. HCT116 **(A)** and LoVo **(B)** cells were treated with 0, 20, and 40 µM GW5074 in the absence or presence of 10 µM sorafenib for 24 h, after which the live cells were stained with 5 mM MitoSOX™Red for 10 min at 37°C and assayed using a flow cytometer. The results are representative of three independent experiments. **p < 0.01 and ***p < 0.001.

A previous study pointed out that C-RAF maintains cell survival by controlling ROS production and Ca^2+^ homeostasis of mitochondria (27). Next, we used the ROS-sensitive dye DCF-DA and the specific mitochondrial superoxide indicator MitoSOX™Red with flow cytometry for monitoring the generation of ROS in cytoplasm and mitochondria of HCT116 and LoVo cells treated with sorafenib, GW5074, and both combined (**Figures 7**, **8**). The generation of cytosolic ROS was significantly enhanced by sorafenib and GW5074, but GW5074 failed to potentiate the capacity of sorafenib at 10 and 40 µM in HCT116 and 40 µM in LoVo cells (**Figures 7A, B**). The generation of mitochondrial ROS was significantly enhanced by sorafenib and GW5074 (**Figures 8A, B**). The combined therapy had a significant additive effect on increasing ROS in mitochondria of HCT116 and LoVo cells. The oxidative capacity is determined by oxidative stress, which highlights the crucial role of antioxidant defenses in the redox homeostasis of the organism. Hence, we analyzed proteins related to oxidative stress, such as NRF2 and HO-1, and antioxidant defense mechanisms, such as catalase, superoxide dismutase 1 (SOD1), SOD2, and SOD3, in HCT116 and LoVo cells. Our Western blotting data showed that sorafenib and GW5074 elevated the level of NRF2 in HCT116 cells, and both combined decreased the levels of SOD1 and SOD2 in HCT116 and LoVo cells (**Figures 7C, D**). A well-known DNA damage biomarker, γH2A.x, was suppressed in the presence of sorafenib-treated HCT116 cells and increased by both combined in LoVo cells.

### The effects of Sorafenib, GW5074, and Both Combined on Mitochondrial Functions in HCT116 and LoVo Cells

**Figure 9:**
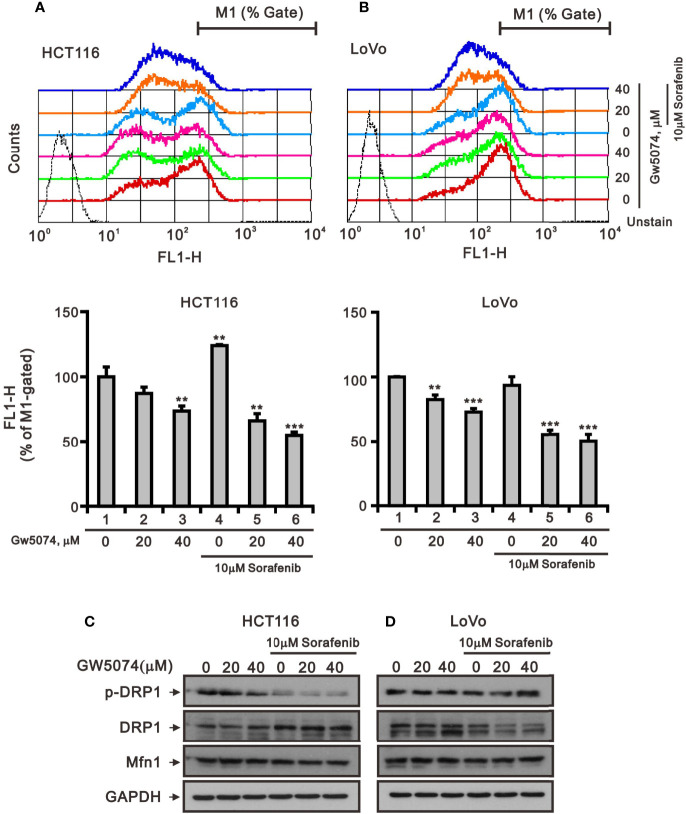
The effects of GW5074 and sorafenib on the mitochondrial fission–fusion transient of HCT116 and LoVo cells. HCT116 **(A)** and LoVo **(B)** cells were treated with 0, 20, and 40 µM GW5074 in the absence or presence of 10 µM sorafenib for 24 h, after which the live cells were stained with 100 nM MitoView™Green for 15 min at 37°C and assayed using a flow cytometer. **(C, D)** Cell lysates were subjected to Western blotting analysis with antibodies against DRP, p-DRP, and Mfn1. GAPDH is a loading control protein. The results **(A, B)** are representative of three independent experiments. **p < 0.01 and ***p < 0.001.

Mitochondria are dynamic organelles that respond to cell stress by continuously undergoing biogenesis, fission, fusion, mitophagy, and motility (28). Mitochondrial fission is necessary for the selective elimination of mitochondria damaged by mitophagy, and mitochondrial fusion enables surviving fragmented mitochondria to return to the mitochondrial network (29). To test whether sorafenib, GW5074, and both combined treatments affected mitochondrial mass *via* the fission-fusion transient, we stained mitochondria with MitoView™Green, a membrane potential independent dye, and analyzed by flow cytometry. The results indicated that GW5074 and both combined treatments significantly reduced mitochondrial mass in HCT116 and LoVo cells (**Figures 9A, B**). Two outer mitochondrial membrane proteins, dynamin-related protein 1 (DRP1) and mitofusin 1 (Mfn1), are involved in the dynamic processes of mitochondrial fission and fusion, respectively (30, 31). The ratio of p-DRP1/DRP1, the biomarker for mitochondrial fission, was reduced by sorafenib, GW5074, and both combined treatments in HCT116 cells (**Figure 9C**), whereas the ratio of p-DRP1/DRP1 was increased by sorafenib and both combined treatments in LoVo cells (**Figure 9D**).

**Figure 10:**
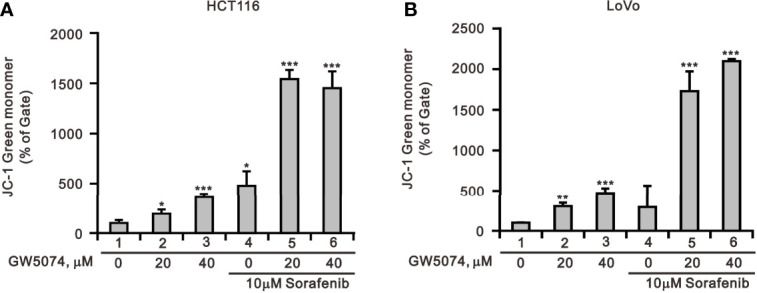
The effects of GW5074 and sorafenib on the mitochondrial membrane potential of HCT116 and LoVo cells. HCT116 **(A)** and LoVo **(B)** cells were treated with 0, 20, and 40 µM GW5074 in the absence or presence of 10 µM sorafenib for 24 h and assayed with JC-1 dye using a flow cytometer. The results are representative of three independent experiments. *p < 0.05, **p < 0.01, and ***p < 0.001.

According to a previous report, each member of the RAF family presents a specific distribution at the level of cellular membranes, and C-RAF is the only isoform that directly targets mitochondria—it plays an important role in mitochondria, regulating the shape and the cellular distribution of mitochondria and making it a target of the combination of sorafenib and GW5074 in some cancers (32). Mitochondrial dysfunction is involved in the induction of apoptosis and is even considered to be the core of the apoptosis pathway (33). Therefore, we used JC-1 dye to examine the disruption of mitochondrial membrane potential after the combination of sorafenib and GW5074 in HCT116 and LoVo cells, which is considered a hallmark of apoptosis. In non-apoptotic cells, stained red JC-1 exists in the form of dimers and accumulates in the form of aggregates in mitochondria. In apoptotic cells, JC-1 exists in the cytoplasm as a monomer and is stained green. The results showed that when HCT116 and LoVo cells were treated alone with GW5074, mitochondrial membrane potential loss levels increased with dose (**Figure 10**). Combination treatment with sorafenib 10 µM dramatically increased the loss of mitochondrial membrane potential. This finding suggests that the combined treatment of sorafenib and GW5074 led to more severe mitochondrial damage in HCT116 and LoVo cells.

**Figure 11:**
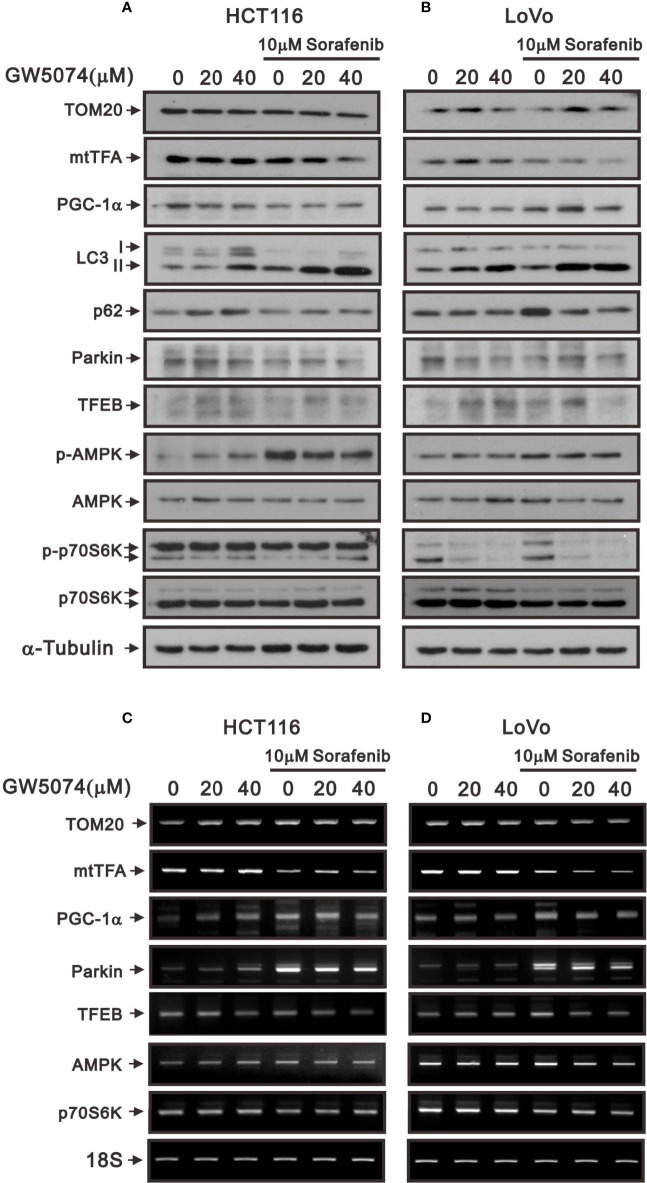
The effects of GW5074 and sorafenib on the mitochondrial autophagy and biogenesis of HCT116 and LoVo cells. HCT116 and LoVo cells were treated with 0, 20, and 40 µM GW5074 in the absence or presence of 10 µM sorafenib for 24 h. **(A, B)** Cell lysates were subjected to Western blotting analysis. α-Tubulin is a loading control protein. **(C, D)** Total RNA were subjected to RT-PCR analysis. 18S is a loading control.

Changes in overall mitochondrial mass represent changes in the balance of mitochondrial biogenesis and mitophagy levels (34). Mitochondrial transcription factor A (mtTFA) and peroxisome-proliferator-activated receptor γ co-activator-1α (PGC-1α) are two key mitochondrial biogenic and respiratory factors for mitochondrial respiratory function (35, 36). Therefore, we subsequently examined the protein levels of mitochondrial biogenesis, respiration, and mitophagy such as PGC-1α, mtTFA, Tom20, parkin, and autophagy using Western blotting analysis. The protein levels of Tom20, mtTFA, and PGC-1α were affected variably by sorafenib and GW5074 in HCT116 and LoVo cells; however, GW5074 had the suppressive effect of sorafenib on these proteins in both cells (**Figures 11A, B**). The mRNA levels of mtTFA and PGC-1α were suppressed by sorafenib which was enhanced by GW5074 (**Figures 11C, D**). Autophagy plays a dynamic tumor-suppressive or tumor-promoting role in different contexts and stages of cancer development. We further examined the biomarker of autophagy, LC3B; the II/I ratios were increased in HCTA116 and LoVo cells treated with sorafenib, GW5074, and both combined (**Figures 11A, B**). An autophagic cargo p62 did not follow the trend in both cell lines. Mitophagy, selective autophagy in mitochondria, is regulated by PINK1 and parkin proteins. The parkin proteins were suppressed with the combination of sorafenib and GW5074 (**Figures 11A, B**), but the mRNAs were enhanced in HCT116 and LoVo cells (**Figures 11C, D**). TFEB (transcription factor E3) is one of the master transcriptional regulators of autophagy and the AMPK-p70S6K pathway is a positive pathway for autophagy. The protein and mRNA levels of TFEB did not show a clear trend. The ratio of p-AMPK/AMPK was elevated by sorafenib, which could be slightly suppressed by GW5074 in both cells; the ratio of p-p70S6K/p70S6K was strongly suppressed by GW5074 in LoVo cells, and the mRNA levels of p70S6K were decreased by sorafenib combined with GW5074 in LoVo cells.

**Figure 12:**
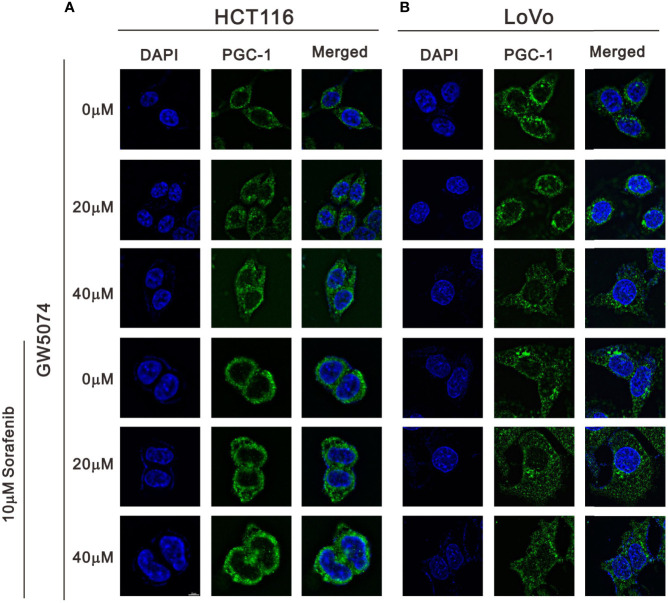
The effects of GW5074 and sorafenib on the expression of PGC-1α in HCT116 and LoVo cells. HCT116 **(A)** and LoVo **(B)** cells were treated with 0, 20, and 40 µM GW5074 in the absence or presence of 10 µM sorafenib for 24 h and were immune-stained with anti-PGC-1α (green) and DAPI (blue). Images were examined under a Leica Thunder microscope with a 100x objective. Scale Bar=10 μm.

**Figure 13:**
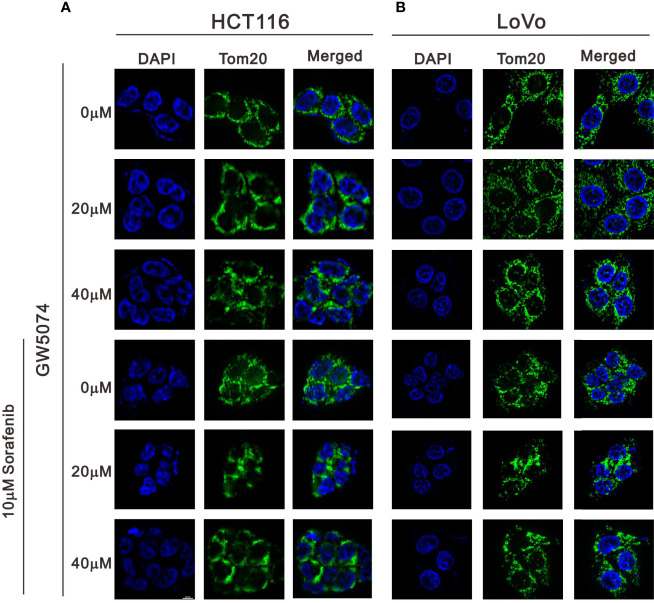
The effects of GW5074 and sorafenib on the expression of Tom20 in HCT116 and LoVo cells. HCT116 **(A)** and LoVo **(B)** cells were treated with 0, 20, and 40 µM GW5074 in the absence or presence of 10 µM sorafenib for 24 h and were immune-stained with anti-Tom20 (green) and DAPI (blue). Images were examined under a Leica Thunder microscope with a 100x objective. Scale Bar=10 μm.

We observed mitochondrial morphology by immunofluorescence staining for PGC-1α and TOM20 with sorafenib, GW5074, and the combined treatment in HCT116 and LoVo cells (**Figures 12**, **13**). The amounts of PGC-1α were elevated by sorafenib and the subcellular distributions were changed by GW5074 and sorafenib (**Figure 12**). More nuclear PGC-1α proteins were observed with the combination of sorafenib and GW5074 in HCT116 and LoVo cells. The subcellular distributions of TOM20 were changed from fragmented to tubular forms by GW5074 and sorafenib (**Figure 13**).

## Discussion

The efficacy of sorafenib target therapy may also be altered by the development of drug resistance and cancer recurrence in CRC. In this study, we combined sorafenib with GW5074 to reduce the dose of sorafenib and enhance its cytotoxicity in two CRC cell lines, HCT116 and LoVo cells. Our findings demonstrate that GW5074 can potentiate the cytotoxicity of sorafenib and dramatically reduce the IC50 dose of sorafenib from 17 and 31 µM to 0.14 and 0.01 µM in HCT116 and LoVo cells, respectively. GW5074, similar to sorafenib, suppressed the cellular proliferation and induced cellular apoptosis and cytosolic ROS, but had no further enhancement on the above-mentioned effects when combined with sorafenib. The synergistic effects of GW5074 and sorafenib were mainly exerted in impacting mitochondrial functions, including ROS generation, membrane potential disruption, and fission–fusion dynamics, which were examined by using the flow cytometry analysis. In summary, the C-RAF inhibitor GW5074 might potentiate the cytotoxicity of the B-RAF inhibitor sorafenib mediated through mitochondrial dysfunctions, suggesting that GW5074 potentially serves as a sensitizer for sorafenib application to reduce the risk of drug resistance of CRC treatment.

pC-RAF^S338^ interacted with pDAPK^S308^ and directed it to become colocalized in the mitochondria (37). A study by Cha showed that sorafenib and GW5074 bound to mitochondrial C-RAF and induced a conformational change to compromise its mitochondrial targeting capability (20). GW5074 and sorafenib combination therapy resulted in the translocation of pC-RAF^S338^ from the mitochondria to the cytoplasm, concomitant with a decrease in mitochondrial membrane potential and an increase in ROS generation in RCC. In CRC, we observed similar effects of GW5074 and sorafenib combination therapy on the disruption of mitochondrial membrane potential and the induction of ROS generation with decreasing amounts of pDAPK^S308^ (data not shown). The cytotoxicity of GW5074 alone was hard to detect in LoVo cells, whereas it could induce mitochondrial ROS generation and the disruption of mitochondrial membrane potential and fission–fusion dynamics. The detailed mechanisms involved in the regulation of mitochondrial function by pDAPK^S308^ and pC-RAF^S338^ and the effectiveness of various mitochondrial functions are worthy of further investigation in CRC.

Sorafenib is a multi-kinase inhibitor with activity against B/C-RAF, B-RAF^V600E^, Flt3, Kit, RET, VEGFR1/2/3, and PDGFRβ (11, 12, 38). Sorafenib is the only first-line therapeutic targeted drug for advanced hepatocellular carcinoma (HCC) (39, 40). Sorafenib resistance is mainly focused on the activation of sorafenib targets and downstream signaling, the regulation of cell proliferative and apoptotic signals, and the epithelial–mesenchymal transition and stemness. Studies have demonstrated that sorafenib induces autophagy, correlated with the reduction in sorafenib sensitivity (25). Research literature has demonstrated that activated ER stress can induce autophagy (41). A recent study demonstrated that melatonin regulates ER stress-induced autophagy to overcome apoptosis resistance and increase the sensitivity to sorafenib in HCC cells (42). Here, compared with sorafenib alone, the combination of GW5074 with sorafenib induced ATF4-CHOP and reduced ATF6 and the ratio of p-eIF2/eIF2 in HCT116 and LoVo cells. In addition, mTOR pathway activation is responsible for the acquisition of resistance to sorafenib in HCC therapy (43). In our study, GW5074 combined with sorafenib reduced the ratio of pAMPK/AMPK in HCT116 and the amount of p62 in LoVo cells. Hence, the combination of GW5074 with sorafenib might mediate through the induction of ER stress and suppression of mTOR activation to decrease the chance of sorafenib resistance in our current study. However, the detailed mechanism should be checked in sorafenib-resistant CRC cell lines.

ROS plays a central role in cell signaling mediated by mitochondria (26). A two-hit working model of sorafenib combined with GW5074 was proposed, where cytosolic translocation of C-RAF/pDAPK^S308^ induces mitochondrial dysfunction to produce ROS (first hit) and triggers PP2A-mediated de-phosphorylation and activation of DAPK (second hit) in RCC (20). Our data on cytosolic and mitochondrial ROS generation suggest that GW5074 synergistically enhances the ability of ROS generation in mitochondria, not in the cytosol, which might be mediated through the disruption of mitochondrial membrane potential. Mitochondria also have an important role in triggering and regulating apoptosis; this synergy effect was not found in our apoptotic Annexin V analysis. The generation of ROS in cells exists in equilibrium with a variety of antioxidant defenses, including SODs (44). We observed the downregulation of SOD1 and SOD2 with the combination of GW5074 and sorafenib in HCT116 and LoVo cells. It is important to elucidate how mitochondrial or cytosolic ROS are involved in the induction of apoptosis using mitochondrial or cytosolic ROS scavengers.

The synergistic effect between sorafenib and GW5074 on the cytotoxicity and mitochondrial functions might be the primary contribution of our current work which also is encouraged by these ongoing clinical trials. However, the limitation of the current study is a lack of a sorafenib-resistant HCT116 or LoVo cell line to verify the synergistic effect of GW5074 on these resistant CRC cells. In addition to DAPK, more phosphorylation status of B-RAF and C-RAF targets by GW5074 and/or sorafenib should be examined to support our findings *via* the inhibitors for B-RAF and C-RAF.

## Data Availability Statement

The original contributions presented in the study are included in the article/supplementary material. Further inquiries can be directed to the corresponding author.

## Author Contributions

Conceptualization, J-MH and S-MH. Methodology, Y-LC. Validation, J-MH and Y-LC. Formal analysis, J-MH. Investigation, J-MH. Resources, C-CH. Data curation, J-MH. Writing—original draft preparation, J-MH. Writing—review and editing, S-MH. Supervision, S-MH. Project administration, C-CH. All authors have read and agreed to the published version of the manuscript. All authors contributed to the article and approved the submitted version.

## Funding

This work was supported by a grant from the Tri-Service General Hospital [TSGH-E-111194 to J-M Hu], Taiwan, Republic of China.

## Conflict of Interest

The authors declare that the research was conducted in the absence of any commercial or financial relationships that could be construed as a potential conflict of interest.

## Publisher’s Note

All claims expressed in this article are solely those of the authors and do not necessarily represent those of their affiliated organizations, or those of the publisher, the editors and the reviewers. Any product that may be evaluated in this article, or claim that may be made by its manufacturer, is not guaranteed or endorsed by the publisher.
